# Investigations into Recycling Zinc from Used Metal Oxide Varistors via pH Selective Leaching: Characterization, Leaching, and Residue Analysis

**DOI:** 10.1155/2015/653219

**Published:** 2015-09-01

**Authors:** Toni Gutknecht, Anna Gustafsson, Christer Forsgren, Christian Ekberg, Britt-Marie Steenari

**Affiliations:** ^1^Industrial Materials Recycling, Department of Chemical Engineering, Chalmers University of Technology, Kemivägen 4, 412 96 Gothenburg, Sweden; ^2^Stena Metall AB, Fiskhamnsgatan 8D, Box 4088, 400 40 Gothenburg, Sweden

## Abstract

Metal oxide varistors (MOVs) are a type of resistor with significantly nonlinear current-voltage characteristics commonly used in power lines to protect against overvoltages. If a proper recycling plan is developed MOVs can be an excellent source of secondary zinc because they contain over 90 weight percent zinc oxide. The oxides of antimony, bismuth, and to a lesser degree cobalt, manganese, and nickel are also present in varistors. Characterization of the MOV showed that cobalt, nickel, and manganese were not present in the varistor material at concentrations greater than one weight percent. This investigation determined whether a pH selective dissolution (leaching) process can be utilized as a starting point for hydrometallurgical recycling of the zinc in MOVs. This investigation showed it was possible to selectively leach zinc from the MOV without coleaching of bismuth and antimony by selecting a suitable pH, mainly higher than 3 for acids investigated. It was not possible to leach zinc without coleaching of manganese, cobalt, and nickel. It can be concluded from results obtained with the acids used, acetic, hydrochloric, nitric, and sulfuric, that sulfate leaching produced the most desirable results with respect to zinc leaching and it is also used extensively in industrial zinc production.

## 1. Introduction

It is the vision for Europe to have market and policy incentives in place by 2020 that will stimulate new innovations in resource efficient production methods with all companies being able to measure their lifecycle resource efficiency [1]. It is with this vision that the importance of this work comes to light. Electrical transmission and distribution equipment such as insulators and protective equipment will become a potentially large source of solid waste suitable for recycling as opposed to landfilling. Recycling of the used varistor material and production waste promotes sustainable production and consumption. Moreover, improving waste management makes better use of resources while encouraging less dependence on imports of raw material [1].

In Sweden, there is an initiative to recycle MOV as opposed to landfilling due to environmental concerns, rising costs of landfilling, awareness of the potential value of the material in the MOV, and the quantity of material available for recycling. In Sweden from 2009 to 2013 over 500 tons of MOV was available for recycling [2]. However, a commercial method for recycling of the MOV is not yet available and the material is at the moment stored or landfilled. If the ZnO was purified and converted to zinc metal the value of the metallic zinc would be over $800,000 USD [3]. The used varistors are primarily not mixed with other types of waste materials but kept in a separate flow which is a good starting point for a recycling process. The authors have not found any literature on recycling of MOVs.

MOVs are made by combining powdered metal oxides of zinc, antimony, bismuth, manganese, nickel, and cobalt. The metal oxide powder is sintered in a process during which three main microstructural phases form: ZnO grains, an antimony-rich phase, and a bismuth-rich intergranular phase [4–6]. The ZnO grain phase is by far the most dominant region due to the MOV being composed mostly of zinc oxide [7]. The antimony-rich phase resulting from sintering and reactions between the metal oxides has been known to include a pyrochlore phase as well as a spinel phase each with different stoichiometry [8, 9]. Pyrochlore is a zinc-bismuth-antimony-oxide (Zn_2_Bi_3_Sb_3_O_12_) [10] and the other a spinel phase containing both a cubic (Zn_2.33_Sb_0.67_O_4_) and an orthorhombic (Zn_7_Sb_2_O_12_) configuration [11]. Initially Sb_2_O_3_ is added to the starting material to decrease the average size of the ZnO grains [5, 9, 12]. The current-voltage behavior of the MOV is attributed mainly to the presence of Bi_2_O_3_ [9] which also strongly alters the sintering behavior by producing a liquid phase with ZnO enabling liquid phase sintering [13–15].

It has been reported in literature that metal oxides such as MnO_2_, NiO, and Co_2_O_3_ and other minor metal oxides may be present in the MOV added to enhance the characteristics of the MOV [4, 5, 12–15]. Typically MOV contains greater than 90 mol% ZnO and around 3 mol% of both Bi_2_O_3_ and Sb_2_O_3_ with the other metal oxides accounting for the remaining 4 mol% [15, 16].

This work investigates the feasibility of selectively leaching zinc from the MOV at a certain pH as an initial step for recovery of secondary zinc. Optimal zinc leaching would avoid coleaching of antimony, bismuth, and other minor metals present in the MOV making the leachate easy to integrate into industrial zinc electrowinning solutions. Industrially sulfuric acid is used in zinc production and it was therefore investigated in this work as well as other acids including acetic acid, which is a weak monoprotic organic acid, nitric acid, and hydrochloric acid.

In general there are two routes available for industrial zinc purification and production. First, a high temperature pyrometallurgical process where activated charcoal is added to the zinc oxide containing material and heated to temperatures above 1000°C at which point zinc is vaporized. The zinc vapor is condensed and collected either as ZnO or impure zinc which is further refined electrolytically [17, 18]. It is known that even low concentrations of antimony in the electrolyte can reduce the current efficiency of zinc electrowinning by nearly 80% [19] and that other impurities such as cobalt, nickel, and manganese can also reduce the current efficiency. Ideally an acid and pH which only leached zinc would be preferred but it is known from other literature data that leaching of antimony and cobalt will probably interfere in leaching of the MOV material [19–21].

The second route is hydrometallurgical purification of ZnO feed material which produces around 80% of the world's zinc [18, 22] and is typically preferred over pyroprocessing due to the effectiveness, process flexibility, and low temperatures. Pyrometallurgical processes are typically energy intensive and often need a dust collection or gas cleaning system [23]. When choosing either a pyroprocess or hydroprocess for recycling MOV the preferred method will have to be economically feasible with respect to the costs of purchasing raw starting materials. There is a plethora of literature reviews and articles on zinc recovery from industrial waste [23] but this is the first of its kind on recovery of zinc from recycled MOV.

## 2. Materials and Methods

### 2.1. Characterization

Identification and composition of the additives in the specific type of MOV investigated needed to be determined as only the major metal oxides: zinc, bismuth, and antimony were known. Additives or impurities (any metal other than zinc or ZnO) in the MOV sample may have an impact on zinc leaching and the eventual electrolytic process. New MOVs approximately 70 mm in diameter and weighing 1000 g were broken up into pieces approximately 2 cm in diameter. An impact mill was used for further particle size reduction. The crushed MOV was mechanically sieved. In leaching experiments material having a particle size smaller than 63 *μ*m was used (100%, −250 Mesh). For leaching smaller particle sizes equate to higher surface area and quicker leaching kinetics are typically observed.

The appearance of the ground and sieved MOV was analyzed with a scanning electron microscope (SEM) with energy dispersive X-ray (EDX) spectroscopic element detection (Hitachi TM 3000 with EDX, Quantax 70) to obtain qualitative data about the elements present and to determine the occurrence and distribution of the components. X-ray powder diffractometry (XRD) (Bruker 2D Phaser) equipped with a characteristic Cu radiation source and a scintillation detector was used to identify crystalline compounds present in the MOV powder. Compound identification was made by comparisons with standards in the Joint Committee of Powder Diffraction Standards database [24].

To determine the metal content in the MOV, complete dissolution of the MOV powder was performed in triplicate using concentrated hydrochloric acid at an elevated temperature. The MOV material (approximately 2.5 g) was heated with 50 mL concentrated hydrochloric acid (37%) at 70 ± 3°C for 12 h while being continuously stirred using a magnetic stir bar. Before determination of metal concentrations, aliquots of the solutions were diluted with a 1 M nitric acid solution, prepared from concentrated stock solution (65%, Suprapur, Merck) and ultrapure water (Milli-Q, Millipore, >18 MΩ/cm). Analysis was done using Inductive Coupled Plasma with Optical Emission Spectrometric detection (ICP-OES) (iCAP 6500, Thermo Fischer). External calibration curves made by dilution of 1 mg/mL standard solutions were used to quantify metal contents.

### 2.2. Leaching

Leaching experiments were started by mixing 0.5 g of powdered MOV and 50 mL of milli-Q water in a straight wall, 150 mL capacity, titration vessel. The vessel was equipped with a pH electrode, a stir bar, and a dosing device connected to a Metrohm 905 Titrando titrator connected to a computer for monitoring and controlling the acid addition. Acid was titrated into the MOV-water mixture resulting in a leachate with a specified pH.

Small aliquots of the leachate were taken at times 0, 2, 10, 30, 60, 120, and 240 minutes in each leaching experiment. The pH was controlled using a silver/silver chloride (Ag/AgCl) glass electrode. Calibration of the pH electrode was done weekly using Metrohmn Ion analysis buffer solutions of pH 4, 7, and 9 while the measured pH value was not corrected to compensate for changes in the ionic strength as the ionic strength of this solution is lower than 1. The temperature of the system was maintained at 25°C  ±  1.

In total four acid solutions were used for the leaching studies: acetic acid (≥99.7%, Sigma Aldrich), hydrochloric acid (37%, Sigma Aldrich), nitric acid (65%, Suprapur, Merck), and sulfuric acid (95.0–98.0%). Leaching experiments were carried out at constant pH of 1, 3, and 5 for each acid solution with the exception of acetic acid in which leaching experiments were carried out having final pH 2, 3, and 5. The acid leaching solutions were not initially prepared to the desired pH. Rather the desired pH was entered into the titration program and a more concentrated acid solution was added to the water-MOV system until the desired pH of the system was reached. The system was stirred so the stagnant layer around the solid particles could be perturbed ensuring mass transport from the liquid in the pores to the outer leachate where the pH and metal concentrations were measured.

In order to determine the concentration of the leached metals as a function of time an aliquot taken at each point of time was centrifuged and diluted with 1 M HNO_3_ for further analysis using ICP-OES. The following metals were analyzed: Bi, Co, Cu, Fe, Mg, Mn, Ni, Sb, and Zn. However, Cu, Fe, and Mg were not detected and are therefore not reported. Leaching experiments were done in triplicate to ensure experimental reproducibility of leaching and leaching equipment and to account for deviation and error propagation in the measurements. The concentrations of metals in the leachates were compared to the concentration of the metals from the complete dissolution experiments allowing for data to be presented as the fraction of each metal leached.

Because each acid has the ability to form complexes with metal ions the speciation of zinc was also considered in each acid solution. The software used for speciation of metal ions in the leachates, PHREEQC [25] using the* minteq* database, provided data on the metal-anion complexes for zinc but did not have information available on complexation of bismuth or antimony. PHREEQC is a computer program used to model equilibrium and dissolution reactions [26]. Concentration of zinc and acid ions in solution at the end of the leaching experiment (pH 1, 3, and 5) were used as input data.

## 3. Results and Discussion

### 3.1. Characterization of MOV

The MOV used in this study was purchased from a commercial varistor production company. The assumption is made that the composition of the varistor does not change over its useful life, at least on the macroscopic scale. On a microscopic (monolayer) scale it has been shown by Stucki et al., 1987 [27], that the oxygen concentration at the interface region between ZnO grain decreases.

Literature suggests that varistors may contain metal additives (in the oxide form) such as cobalt, chromium, copper, magnesium, manganese, nickel, sulfur, antimony, titanium, tungsten, and yttrium [4, 9, 12–14]. However, the dissolved MOV investigated only contained, in detectible amounts, the metals listed in Table 1 given in weight percent (wt%) and mol% of each metal along with the standard deviation of the measurements.

**Figure 1:**
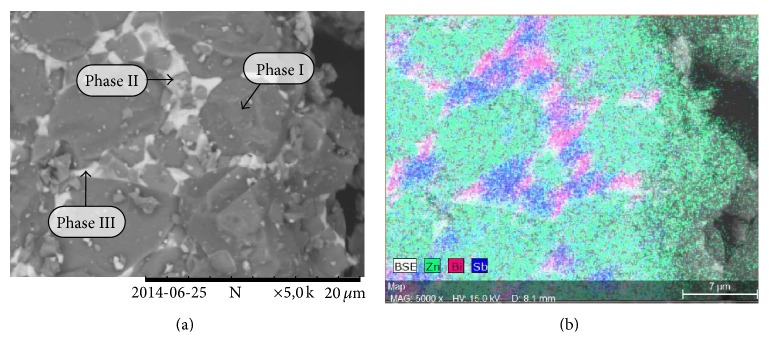
(a) SEM micrograph of pulverized MOV prior to leaching depicting three phases present: (I) ZnO grains, (II) Sb-rich phase, and (III) Bi-rich phase. (b) EDX map of varistor material seen in Figure 1(a) with zinc, bismuth, and antimony-rich phases.

A SEM micrograph of the pulverized (particle size less than 63 *μ*m) MOV is shown in Figure 1(a). From this micrograph three phases can be seen within the MOV. Phase I, the most dominant region, was that of the zinc oxide grains. Phase II was the small particles around the zinc oxide, most likely the antimony-rich phase which, according to literature, includes two phases: pyrochlore and spinel. Phase III was the white bismuth-rich phase. An elemental map was acquired using the EDX detector and is shown in Figure 1(b). The green area depicts the ZnO grains, the purple area is the antimony-rich phase and the pink area is the bismuth-rich phase.

The microstructure of the MOV is polycrystalline making it somewhat complicated to analyze the composition, each phase having different dopants, dopant concentrations, shape, and size. Separation by recycling of the individual metals from the Sb-rich phases may be more complex than leaching of metal ions from the metal oxides. Eventually this may lead to reduced yield and slower kinetics during leaching compared to whether only pure metal oxides had been present. However, from Figure 1(a) it can be seen that the zinc oxide phase is the dominating phase and leaching of zinc is of main importance in this study.

**Figure 2:**
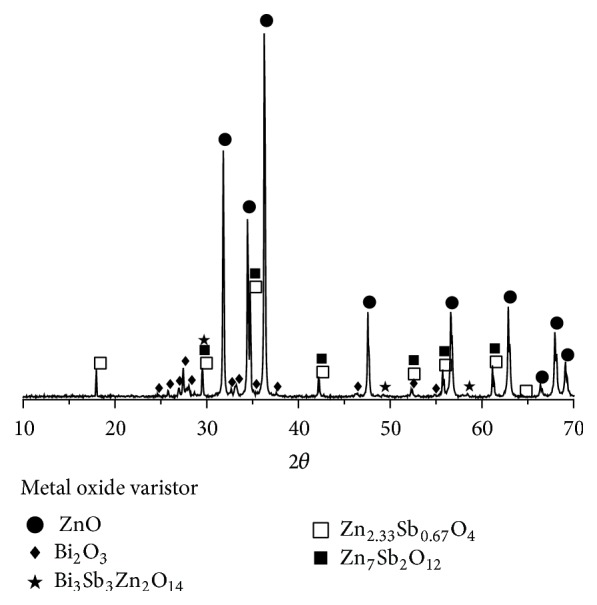
XRD spectra of the MOV showing peaks for ZnO (●), Bi_2_O_3_ (◆), Zn_2.33_Sb_0.67_O_4_ (□), Zn_2_Bi_3_Sb_3_O_14_ (★), and Zn_7_Sb_2_O_12_ (■).

The result from qualitative mineralogical analysis of the MOV using XRD was a spectrum as shown in Figure 2. Peaks correlating to ZnO (●), Bi_2_O_3_ (◆), Zn_2.33_Sb_0.67_O_4_ (□), Zn_2_Bi_3_Sb_3_O_14_ (★), and Zn_7_Sb_2_O_12_ (■) are labeled. Peaks for compounds containing cobalt, manganese, and nickel oxides are not visible due to their low concentrations in the MOV. The majority of peaks shown in Figure 2 are due to the ZnO XRD pattern. There was no peak correlation for antimony oxide confirming that antimony is present in the spinel (Zn_2.33_Sb_0.67_O_4_ and Zn_7_Sb_2_O_12_) or pyrochlore (Zn_2_Bi_3_Sb_3_O_14_) phases. Some peaks correspond to multiple compounds and are labeled accordingly.

### 3.2. Leaching

By leaching the MOV in oxidizing acids (nitric and sulfuric acids), a nonoxidizing acid (hydrochloric acid), and a weak acid (acetic acid) it was expected that a clearer picture of the leaching behavior of zinc, bismuth, and antimony would be determined.

#### 3.2.1. Acetic Acid

**Figure 3:**
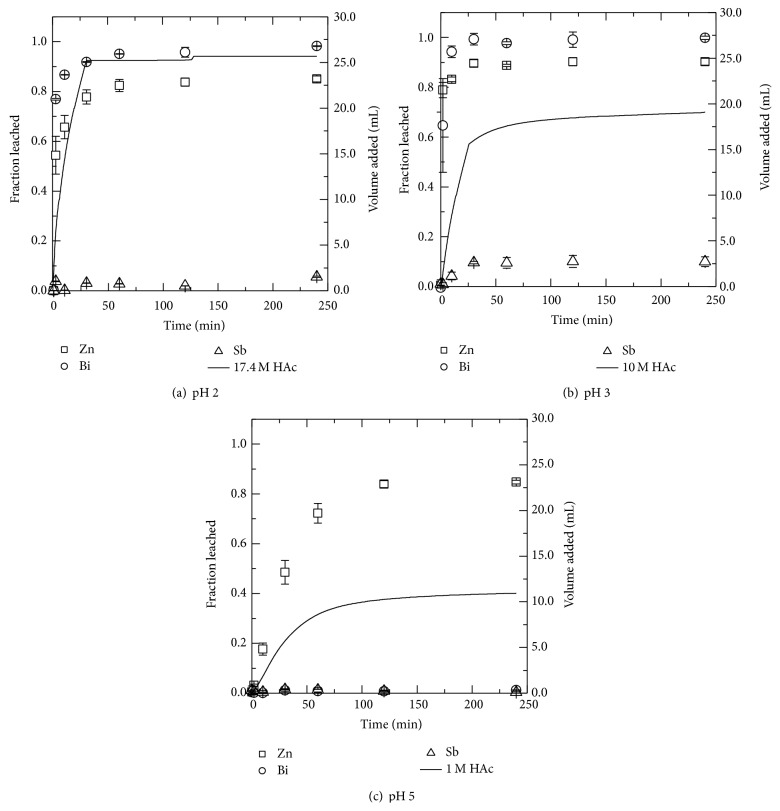
The leached fraction as given by the left ordinate for zinc (□), bismuth (○), and antimony (∆) from MOV in (a) pH 2, (b) pH 3, and (c) pH 5 solutions. The volume of acetic acid (HAc) added is shown as a solid black line corresponding to the right ordinate.

Acetic acid (HAc) was very effective for the leaching of zinc from MOV, as shown in Figure 3. The leached fraction of zinc (□), bismuth (○), and antimony (∆) is shown on the left ordinate while the right ordinate along with the solid line shows the amount of the HAc solution added to the MOV-water slurry to obtain the desired pH. When using HAc solutions at pH higher than 2, up to 90% of the zinc was leached within 4 hours as shown in Figure 3(b) for pH 3 and Figure 3(c) for pH 5. Acetic acid was also effective for leaching bismuth (Figure 3(a)) but to a much lesser extent antimony where ≤20% of Sb was leached in pH 2 solution. The results show that Zn can be selectively leached with no coleaching of Sb or Bi by using an acetic acid leaching solution with pH 5.

Speciation of zinc, regardless of the pH in the range used here, was approximately 44%  Zn(O_2_CCH_3_)_2_, 24% Zn^2+^, 21%  Zn(O_2_CCH_3_)^+^, and 11%  Zn(O_2_CCH_3_)_3_^−^. The dominant bismuth ions are most likely BiOH^2+^, BiO^+^, or a bismuth oxide acetate complex and not Bi^3+^ based on the pH of the solution [28].

As for the other metals present in the MOV, over 90% of the cobalt was leached in the pH 2 solution with the amount of cobalt leached decreasing with increasing pH. Nickel and manganese were both leached around 40% in pH 2 solutions and showed the same trend as cobalt, of decreased leaching with increasing pH.

#### 3.2.2. Hydrochloric Acid

**Figure 4:**
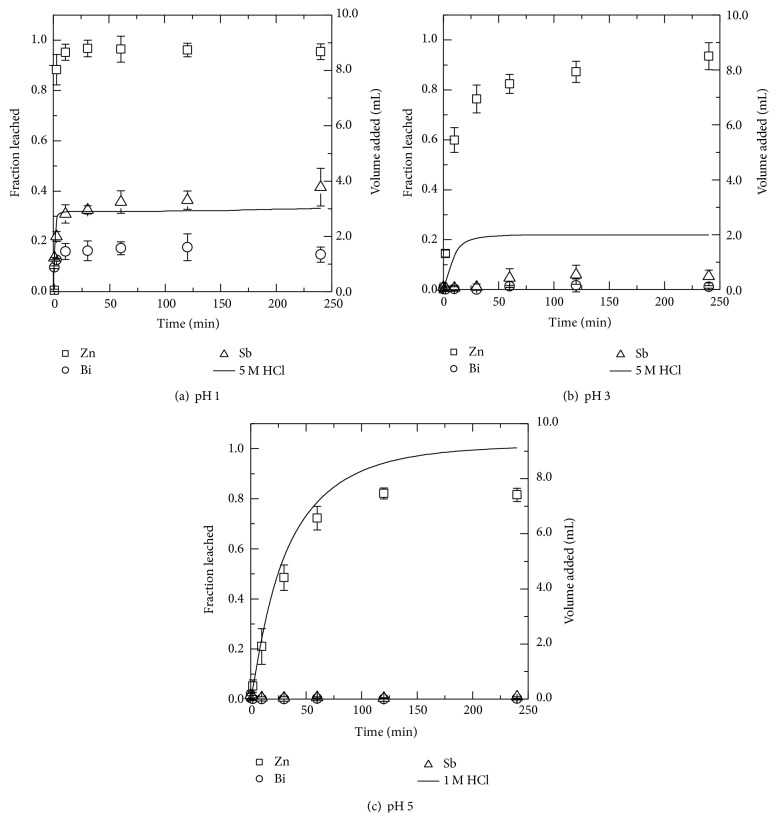
The leached fraction as given by the left ordinate for zinc (□), bismuth (○), and antimony (∆) from MOV in (a) pH 1, (b) pH 3, and (c) pH 5 solutions. The volume of hydrochloric acid (HCl) added is shown as a solid black line corresponding to the right ordinate.

Leaching with pH 1 hydrochloric acid (HCl) solution yielded 95.5 ± 3.1% leaching of zinc from the MOV while it was much more difficult to leach bismuth (20 ± 10%) and antimony (37 ± 6%) at the same pH. However, the results show that zinc can be selectively leached leaving both bismuth and antimony in the MOV residue by using hydrochloric acid leaching solution with a pH higher than 1. Results for hydrochloric acid leaching are shown in Figure 4(a) for pH 1, Figure 4(b) for pH 3, and Figure 4(c) for pH 5.

In HCl solutions with pH greater than 1, Sb_2_O_3_ is not soluble and therefore remains as a solid which is consistent with literature [25]. For Bi_2_O_3_ the dominant species are predicted using *E*_*h*_-pH diagrams to be Bi^3+^ at pH < 2 whereas at pH values > 2 dominant species can be either BiOH^2+^, BiO^+^, or a bismuth oxychloride complex depending on pH [28]. However, from the present results it appears that the oxides of Bi and Sb present in the MOV are only soluble in hydrochloric acid solutions when the pH is higher than 1. This may be due to formation of nonporous and amorphous sintered phases for which the leaching of constituent metal ions is physically hindered. As was shown in Figure 1 bismuth is mainly present as a sintered phase between the ZnO grains.

The speciation of zinc in hydrochloric acid solutions as calculated by PHREEQC indicates that Zn^2+^ is the most dominant species in the hydrochloric acid based leachates obtained here. In the pH 5 hydrochloric solution, Zn^2+^ accounted for approximately 92% of all zinc species but as the pH of the acidic leachate decreased to pH 1 the free Zn^2+^ concentration in solution decreased due to the formation of zinc-chloride complexes. Other zinc-chloride species predicted to be present in pH 1 chloride solutions include ZnCl^+^ (11%), ZnCl_2_ (2%), ZnCl_3_^−^ (0.3%), and ZnCl_4_^−2^ (1.5%).

Not only were hydrochloric acid solutions efficient for zinc leaching, they also worked relatively well for the leaching of manganese, nickel, and especially cobalt. In pH 1 hydrochloric acid solution the percent of cobalt leached was 86% whereas close to 70% and 62% of nickel and manganese, respectively, were leached. Thus, HCl leaching did not give a selective leaching of zinc.

#### 3.2.3. Nitric Acid

**Figure 5:**
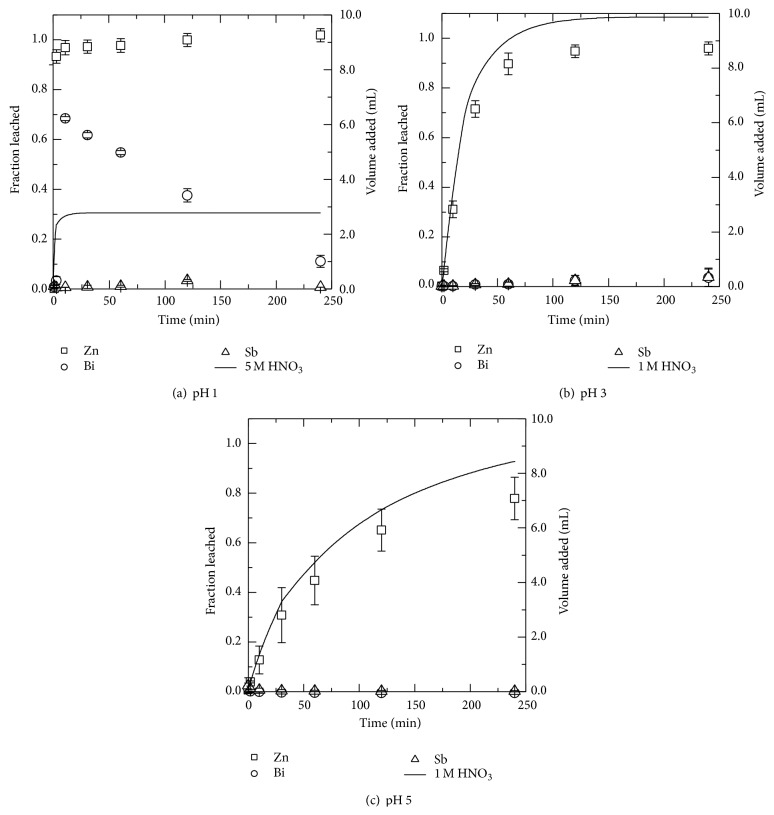
The leached fraction as given by the left ordinate for zinc (□), bismuth (○), and antimony (∆) from MOV in (a) pH 1, (b) pH 3, and (c) pH 5 solutions. The volume of nitric acid (HNO_3_) added is shown as a solid black line corresponding to the right ordinate.

Leaching of MOV in pH 1, 3, and 5 nitric acid (HNO_3_) solutions yielded results as shown in Figure 5 for zinc, bismuth, and antimony. For selective leaching of zinc, pH 1 nitric acid solutions work well due to the high leaching rate for zinc and low leaching of bismuth and antimony. Bismuth showed an atypical leaching behavior as seen in Figure 5(a). Shkol'nikov has reported [29] precipitation of bismuth(III) hydroxy salts near an approximate pH of 1.6 and such a reaction may explain the leaching behavior of bismuth(III) in nitrate solutions. The hydrolyzable bismuth(III) cations have been predicted by thermodynamic calculations to be in solution in more acidic conditions [29]. In pH 5 solutions leaching of alkaline components still occurred at the end of the 4-hour experiment.

Less than 35% of the manganese content was leached from the MOV in the pH 1 nitric acid solution, while 50 and 76% of the nickel and cobalt, respectively, were leached at the same pH. Lower amounts of all these metals, manganese, nickel, and cobalt, were leached at lower concentrations of nitric acid, that is, pH 3 and 5.

#### 3.2.4. Sulfuric Acid

**Figure 6:**
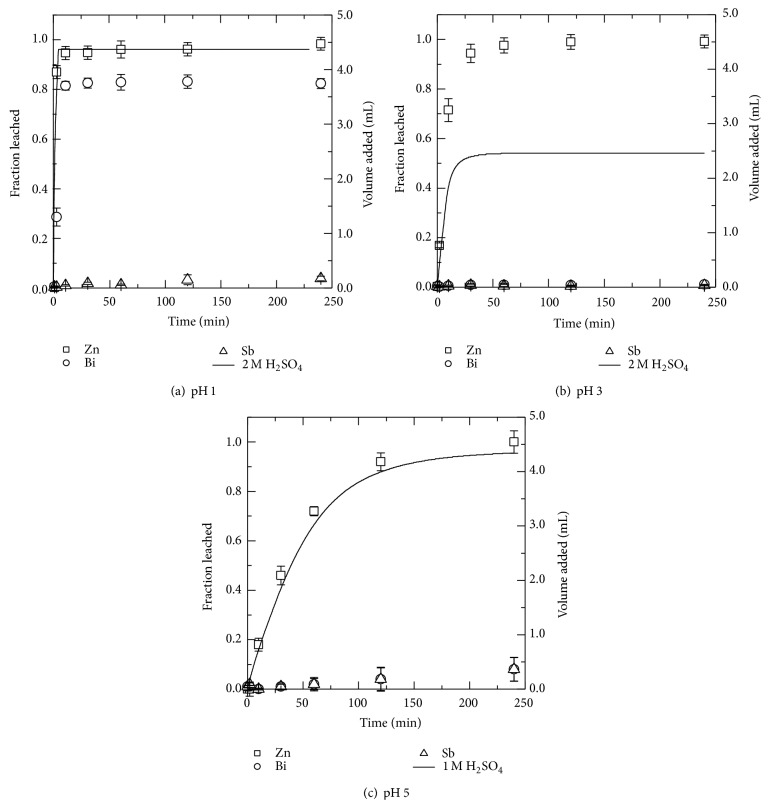
The leached fraction as given by the left ordinate for zinc (□), bismuth (○), and antimony (∆) from MOV in (a) pH 1, (b) pH 3, and (c) pH 5 solutions. The volume and concentration of sulfuric acid (H_2_SO_4_) added is shown as a solid black line corresponding to the right ordinate.

Leaching of MOV in sulfuric acid solutions with pH 1, 3, and 5 gave results as shown in Figures 6(a), 6(b), and 6(c), respectively. Unlike the previously described acid leaching experiments carried out in this work, sulfuric acid solutions were able to leach all Zn at each pH level tested. Increasing the pH from 3 to 5 will not change the percent of Zn leached but rather the time needed for leaching will be longer. It seems to be feasible to use pH 3 solutions to selectively leach zinc while avoiding coleaching of antimony and bismuth. Bismuth is leached when using pH 1 solution and the dominant species should be Bi^3+^ based on the *E*_*h*_-pH diagrams [28]. In these conditions less than 5% of antimony was leached which is consistent with published data saying that oxidizing, acidic solutions should not react with Sb_2_O_3_ [28].

PHREEQC calculations showed that approximately 65% of the zinc in the pH 1 leachate occurred as Zn^2+^ with the remaining 35% of zinc in solution as ZnSO_4_ soluble complex. The pH increased the fraction of zinc as Zn^2+^ ions decreased 55% for pH 5.

Impurities in the zinc leachate include cobalt of which approximately 65% was leached in all solutions investigated. Manganese and nickel were approximately 25% leached in pH 1 solution, 17% at pH 3 solution, and 27% at pH 5. It is not known what causes a lower leaching fraction in pH 3 solution but it could be due to a change in speciation or precipitation of the metals to secondary compounds.

As shown it was possible to selectively leach Zn from the MOV without significant coleaching of bismuth and antimony by selecting a suitable pH, mainly higher than 3 in all acids investigated here. It was not possible to leach zinc without coleaching of manganese, cobalt, and nickel. However, such minor contaminations can be removed before electrowinning of zinc by cementation.

### 3.3. Analysis of Leaching Residue

**Figure 7:**
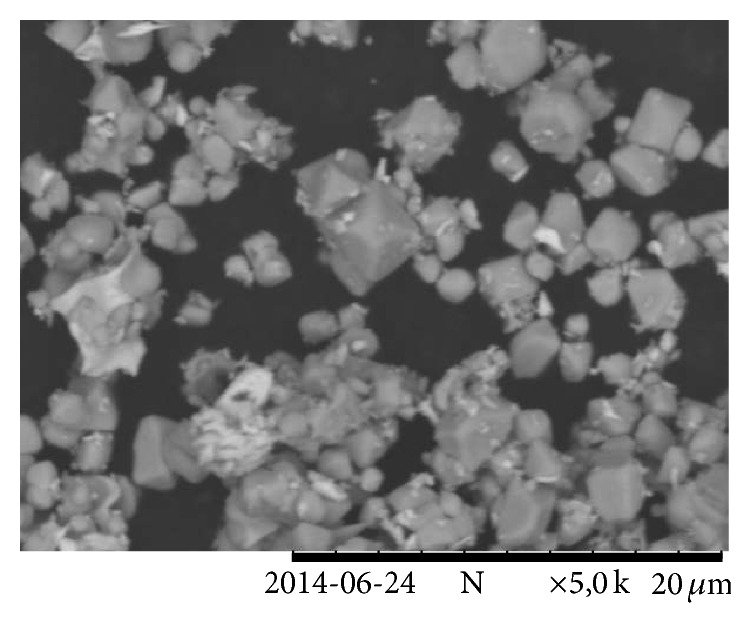
SEM micrograph of pulverized MOV after leaching in pH 1 sulfuric acid solution for 240 minutes. The Sb-rich phase remains along with some undissolved Bi-rich phase.

It was concluded that sulfate leaching produced the most desirable results with respect to zinc leaching and coleaching of other metals ions as well as its extensive use in industrial zinc production. It was also important to determine if zinc leaching was due to bulk leaching of the ZnO grain or if the zinc within the pyrochlore and spinel phases was also leached thus destroying the spinel phase and liberating antimony. The insoluble residue remaining after leaching of the MOV in a pH 1 sulfuric acid solution for 240 minutes is shown in Figure 7. This specimen corresponds to the leaching data in Figure 6(a) where nearly all of the zinc, 80% of the bismuth, and very little antimony had been leached from the MOV. In Figure 7 the dominating structures present are the antimony-rich phases. It occurs in particles of approximately 2 *μ*m in diameter with some residual, undissolved bismuth-rich white phase attached. The SEM micrographs in Figures 1 and 7 illustrate the before and after experimental leaching data of Figure 6.

**Figure 8:**
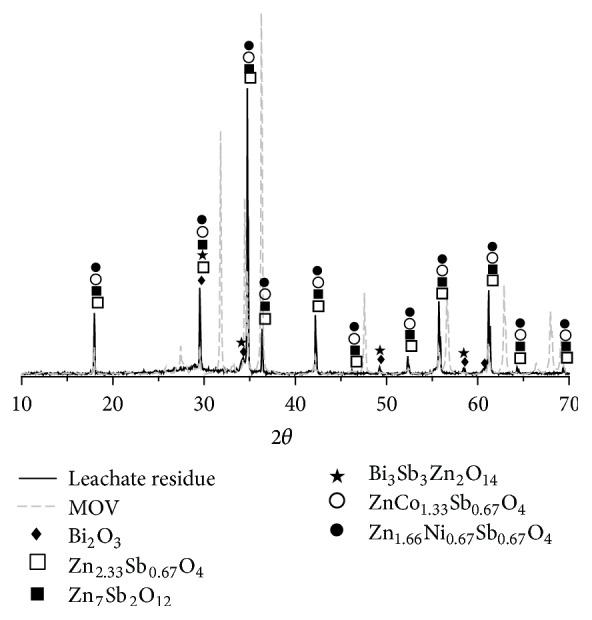
XRD spectrum (—) of leaching residue (pH 1, sulfuric acid) compared to XRD spectrum of nonleached starting material (- - -). Chemical compounds are represented as follows: Bi_2_O_3_ (◆), Zn_2.33_Sb_0.67_O_4_ (□), and Zn_7_Sb_2_O_12_ (■).

XRD analysis results for the pH 1 sulfuric acid leaching residue (Figure 6(a)) are shown in Figure 8 as the solid black line (—). The majority of the peaks can be identified as originating from antimony containing compounds, such as Zn_2.33_Sb_0.67_O_4_ (□), Zn_7_Sb_2_O_12_ (■), ZnCo_1.33_Sb_0.67_O_4_ (○), and Zn_1.66_Ni_0.67_Sb_0.67_O_4_ (●). The four aforementioned chemical compounds all share the same peaks and are all possibly present in the MOV. It might be possible that the concentrations of the minor metals (Co, Ni, and Mn) in the leaching residue identify the presence of some compounds containing Ni, Co, and Mn. However, XRD only suggest the presence is possible not that the compound is actually in the sample.

Also present in the MOV are Zn_2_Bi_3_Sb_3_O_14_ (★) and Bi_2_O_3_ (◆) both having identical peaks. It is most logical based on characterization and literature data that pyrochlore (Zn_2_Bi_3_Sb_3_O_14_) and spinel both cubic (Zn_2.33_Sb_0.67_O_4_) and orthorhombic (Zn_7_Sb_2_O_12_) as well as the as well as the residual Bi_2_O_3_ are present residual Bi_2_O_3_ are present in the sample. It is also probable to have the presence of cobalt, nickel, and manganese in the sample; however the chemical form of those metals is not known. The presence of minor metal oxides is typical of sintered material. The spectrum for the starting material contained prominent peaks for ZnO whereas the appearance of ZnO peaks in the leaching residue was nonexistent. The XRD result also shows that it will be difficult to solubilize the zinc that is present in the combined zinc-antimony oxides.

To summarize, in total four acids were investigated each at three different pH levels. Typically pH 1, pH 3, and pH 5 were used except in the case of acetic acid where pH 1 was difficult to obtain and pH 2 was used instead. Acetic acid leaching results show that selective leaching of zinc from the MOV with respect to bismuth and antimony can be achieved using leaching solution with a pH 5. However in pH 5 acetic acid solutions some bismuth (1.3%  ±  0.1) was leached. In hydrochloric acid solutions zinc can be successfully selectively leached from bismuth and antimony in pH 5 solutions. Similar results for selective leaching of zinc occur in nitric acid solutions with no bismuth or antimony detected in pH 5 solutions. With acetic, hydrochloric, and nitric acid the percent of zinc leached decreased with increasing pH.

For acetic acid nearly 90% of the zinc was leached at pH 2, 3, and 5 while all zinc could be leached using hydrochloric, nitric, and sulfuric acid at pH 1. Increase in pH 5 in hydrochloric and nitric acid solutions resulted in lower zinc leaching with approximately 82% and 78% zinc leached, respectively. Minor metal coleaching at pH 5 is summarized in Table 2. From this data it is shown that leaching with HNO_3_ gives the lowest coleaching percentage of the minor metals present in the MOV but industrially it is not used in zinc production. When comparing sulfuric acid with the others it performs well. The amount of cobalt coleached was around 66% while Mn and Ni were coleached at 27% and 25%, respectively. The minor metal impurities would have to be removed before Zn electrowinning.

Finally, leaching in sulfuric acid solutions was highly effective for zinc leaching at each of the three pH investigated. Leaching at pH 3 resulted in a leachate pure of antimony and bismuth; however the minor metals given in Table 2 will require further purification of the leachate. It is recommended that this acid be used in recycling of the MOV because of the widespread industrial use of sulfate solutions in zinc electrowinning.

Regardless of the leachate used acetic, hydrochloric, nitric, or sulfuric acid further purification of the leachate is required if it is to be used in the zinc electrowinning process. A purification method such as cementation would be effective for removing antimony, bismuth, nickel, and cobalt from the leaching solution. Antimony has the added benefit of being an activator in cementation by increasing the kinetics of the cementation reaction [30].

Speciation modeling using PHREEQC of the zinc in each acid demonstrated that the most prominent form of Zn is Zn^2+^. This is important because the state of the zinc and knowledge of its complexes can affect further zinc recycling steps such as cementation or electrowinning.

## 4. Conclusions

This work set out to determine whether it is possible to separate metal components of the MOV via pH selective leaching in acetic, nitric, hydrochloric, and sulfuric acidic solutions having pH 1, 3, and 5. Initially the composition of the MOV was determined in order to quantify the metals within the MOV. The MOV contains simple oxides such as zinc oxide but it also contains more complex oxides such as Zn_7_Sb_2_O_12_ as shown using the SEM and XRD.

Experimental data showed that lower pH acid solutions gave higher percent of zinc leaching except for the case where H_2_SO_4_ was used and zinc was shown to be fully leached at pH 5 and below. It was possible to selectively leach Zn from the MOV without significant coleaching of bismuth and antimony by selecting a suitable pH, mainly higher than 3 in all acids investigated here. It was not possible to leach zinc without coleaching of manganese, cobalt, and nickel. Even though these metals are present in small amounts in the leachate production of pure zinc metal will require their removal. Sulfuric acid leaching is also preferred because nearly 80% of zinc is produced by electrowinning in sulfate solutions.

This investigation concludes that either acetic, nitric, hydrochloric, or sulfuric acid solutions at pH 5 can be used to selectively leach zinc from the MOV without significant coleaching of antimony or bismuth. However, the efficiency of zinc leached decreases with increasing leaching pH except in the case of sulfate solution. Regardless of the pH in sulfate leaching 100% of the zinc in the MOV was leached making this the ideal selective leaching solution for leaching zinc from MOV. Selective zinc leaching with respect to minor metals such as cobalt, nickel, and manganese could not be successfully done with the acids and pH range under investigation in this study.

## Figures and Tables

**Table 1:** Chemical composition of MOV.

Metal oxide	mol %	wt %
Bi_2_O_3_	2.34 ± 0.06	5.1 ± 0.1
Co_2_O_3_	1.16 ± 0.03	0.94 ± 0.02
MnO_2_	0.76 ± 0.02	0.52 ± 0.01
NiO	0.89 ± 0.02	0.79 ± 0.02
Sb_2_O_3_	3.21 ± 0.08	4.4 ± 0.1
ZnO	91.6 ± 3.3	88.2 ± 3.1

**Table 2:** Percentage of minor metals coleached with zinc in pH 5 leaching solutions.

Acid	Co	Mn	Ni
CH_3_COOH	74.5 ± 0.4	23.4 ± 0.1	19.6 ± 0.3
HCl	72.6 ± 5.5	23.1 ± 2.9	18.5 ± 0.9
HNO_3_	63.3 ± 0.7	19.7 ± 0.3	17.0 ± 0.2
H_2_SO_4_	66.4 ± 3.5	27.1 ± 5.0	24.8 ± 4.6
